# Serial measurements of NT-proBNP are predictive of not-high-dose anthracycline cardiotoxicity in breast cancer patients

**DOI:** 10.1038/bjc.2011.439

**Published:** 2011-11-08

**Authors:** S Romano, S Fratini, E Ricevuto, V Procaccini, G Stifano, M Mancini, M Di Mauro, C Ficorella, M Penco

**Affiliations:** 1 Cardiology, Department of Internal Medicine and Public Health, University of L'Aquila, Piazzale S. Tommasi 1, Coppito -- L'Aquila 67010, Italy; 2 Oncology, Department of Experimental Medicine, University of L'Aquila, L'Aquila, Italy

**Keywords:** anthracyclines, brain natriuretic peptide, cardiac biomarkers, cardiotoxicity, N-terminal pro-brain natriuretic peptide

## Abstract

**Background:**

The aim of this study was to assess the value of N-terminal pro-brain natriuretic peptide (NT-proBNP) in predicting late cardiotoxicity in patients treated with not-high-dose chemotherapy (NHDC), and to compare the predictive value of NT-proBNP and cardiac troponin I (cTnI).

**Methods:**

In 71 patients undergoing NHDC with anthracyclines, NT-proBNP and cTnI levels were measured before and 24 h after each NHDC cycle. Left ventricular (LV) function was assessed by echocardiography at baseline, every two NHDC cycles, at the end of chemotherapy, and at 3-, 6- and 12-month follow-up.

**Results:**

During NHDC, only NT-proBNP showed abnormal values. According to NT-proBNP behaviour, patients were divided into two groups: group A (*n*=50) with normal (*n*=23) or transiently elevated NT-proBNP levels (*n*=27), and group B (*n*=21) with persistently elevated NT-proBNP levels. At follow-up, LV impairment was significantly worse in group B than in group A. %Δ (baseline–peak) NT-proBNP was predictive of LV impairment at 3-, 6- and 12-month follow-up, with a cutoff of 36%.

**Conclusion:**

Serial measurements of NT-proBNP may be a useful tool for the early detection of patients treated with NHDC at high risk of developing cardiotoxicity.

Anthracyclines are powerful chemotherapy agents that are widely used for the treatment of solid and haematological malignancies in both adults and children. Their introduction has led to the successful treatment of several forms of cancer and contributed to reducing mortality and increasing long-term survival.

Notwithstanding this, the use of anthracyclines is limited by dose-dependent cardiotoxicity (Shan *et al*, 1996; Singal and Iliskovic, 1998; Hequet *et al*, 2004; Yeh and Bickford, 2009). Several patients show early or long-term cardiac abnormalities, such as impaired left ventricular (LV) function and/or cardiomyopathy, which may result in overt heart failure and cardiac death. In a series of >5000 long-term cancer survivors treated with chemotherapy agents, the probability of death after relapse was the same as after cardiac complications due to chemotherapy (Schultz *et al*, 2003).

Left ventricular ejection fraction (LVEF), as assessed by echocardiography, is the most commonly used parameter to evaluate LV function in cancer patients undergoing chemotherapy. However, LVEF measurements are not sensitive for the early detection of pre-clinical cardiac disease and are affected by changes in contractility, preload and afterload (Pai and Nahata, 2000; Gharib and Burnett, 2002; Yeh and Bickford, 2009). This emphasises the need for non-invasive, highly sensitive and inexpensive diagnostic tools for the early identification of high-risk patients.

Several studies suggested that cardiac biomarkers may be useful for the identification of patients at high risk of developing cardiotoxicity. The most robust results were obtained using cardiac troponin I (cTnI) (Cardinale *et al*, 2000, 2004; Sparano *et al*, 2002; Adamcova *et al*, 2005; Kilickap *et al*, 2005), whereas the role of natriuretic peptides remains less clear (Bauch *et al*, 1992; Suzuki *et al*, 1998; Meinardi *et al*, 2001, 2002; Sandri *et al*, 2005; Germanakis *et al*, 2006; Kouloubinis *et al*, 2007).

The majority of studies included inpatient cohorts undergoing high-dose chemotherapy (HDC), where blood samples for the determination of cardiac markers can be collected more easily. Only few studies reported the measurement of cardiac markers in outpatients undergoing not-high-dose chemotherapy (NHDC). Although this subset of patients represents the vast majority in several varieties of cancer, information is lacking about the value of cardiac markers as predictors of LV dysfunction.

The aim of our study was (i) to assess N-terminal pro-brain natriuretic peptide (NT-proBNP) increase and behaviour in predicting late cardiotoxicity in patients treated with NHDC and (ii) to compare the predictive value of NT-proBNP and cTnI, the latter being a well-established predictor of cardiotoxicity.

## Materials and methods

### Study population

Ninety-two consecutive breast cancer patients undergoing NHDC with anthracyclines from February 2007 to February 2008 were initially screened for this study. Study eligibility criteria were age between 18 and 75 years, a good performance status (⩽2 on ZUBROD-ECOG-WHO scale), serum creatinine <1.5 mg dl^–1^, normal hepatic function. The following exclusion criteria were applied: history of coronary artery disease, haemodynamically significant valvular heart disease, LVEF <50%, immunological diseases or other serious diseases limiting life expectancy (decompensated diabetes, renal failure, etc.). Patients who had received chemotherapy or local radiotherapy before or during the study period were also excluded. Eleven patients did not match the inclusion/exclusion criteria. In addition, seven patients were excluded because of poor echocardiographic image quality rendering evaluation of LV function unfeasible, and three patients were excluded because of drop-out after the first or second blood draw. Therefore, the final study population consisted of 71 patients, of which 27 had metastatic breast cancer.

Thirty-four patients (48%) were treated with liposome-encapsulated doxorubicin (40–50 mg m^–2^) and docetaxel (50 mg m^–2^) for 6 cycles (every 14 days, dose-dense protocol), and 37 patients (52%) were treated with epirubicin (90 mg m^–2^) in combination with fluorouracil and cyclophosphamide (FEC protocol) for 6 cycles (every 21 days). Patients treated with liposome-encapsulated doxorubicin received a median cumulative doxorubicin dose 300 mg m^–2^. Among FEC-treated patients, dose intensity of epirubicin was >80% and up to 540 mg m^–2^ cumulative dose.

All patients gave their written informed consent. The investigation conformed with the principles outlined in the Declaration of Helsinki and was approved by the local ethics committee.

### Echocardiography

Echocardiographic examination was performed with a commercially available imaging system (MyLab 30, EsaoteBiomedica, Genoa, Italy). Data acquisition was performed with a 3.5-MHz transducer in the parasternal and apical views (standard 2- and 4-chamber images). Two-dimensional and Doppler images were obtained during breath hold and stored in cineloop format from three consecutive beats; then the average values of LV volumes and LVEF were reported.

LV end-diastolic (LVEDV) and end-systolic volumes (LVESV) and LVEF were derived from the apical biplane modified Simpson's rule algorithm. All echocardiograms were interpreted by two experienced cardiologists who were blinded to the patient history and laboratory results; inter-observer and intra-observer variability were 8.7% and 7.9%, respectively.

### Laboratory examination

Blood samples were taken after 5 min of supine rest. NT-proBNP was measured using the commercially available Elecsys proBNP sandwich immunoassay on an Elecsys 2010 (Roche Diagnostics, Mannheim, Germany) for quantitative determination of the peptide in serum and plasma. Intra- and inter-assay variability coefficients were 2.5% and 3.1%, respectively. The upper reference limits of NT-proBNP were 153 ng l^–1^ for patients aged ⩽50 years and 222 ng l^–1^ for patients aged >50 years, according to the manufacturer's guidelines. cTnI concentrations were determined by a fluorometric enzyme immunoassay analyser (Stratus CS Acute Care, Dade Behring, Deerfield, IL, USA); the cutoff levels were 5 and 0.08 ng ml^–1^, respectively. All positive samples were immediately retested for confirmation.

### Study protocol

**Figure 1:**
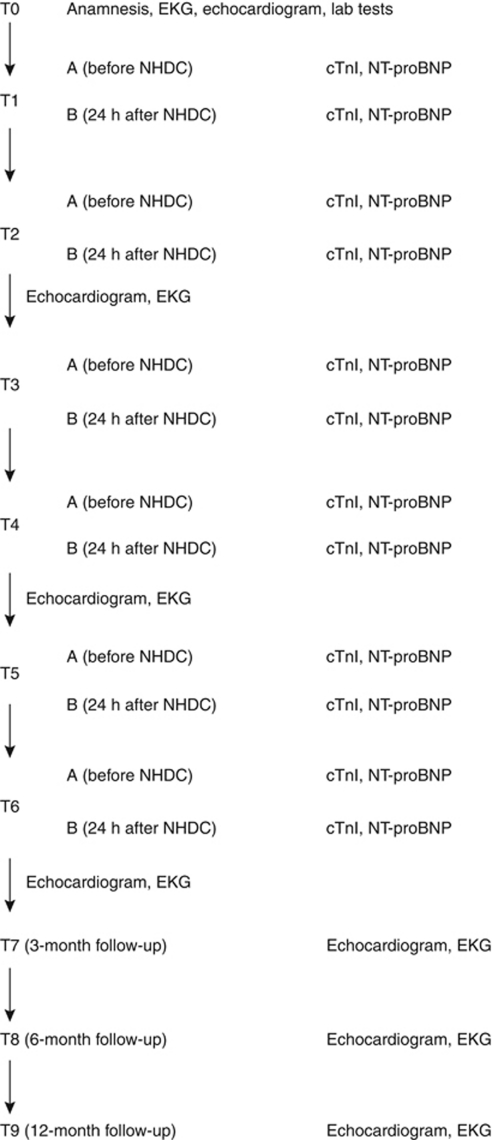
Flowchart of the study protocol: T0=1 week before the beginning of the study; T1=first NHDC cycle; T2=second NHDC cycle; T3=third NHDC cycle; T4=fourth NHDC cycle; T5=fifth NHDC cycle; T6=sixth NHDC cycle.

All patients underwent complete anamnestic, clinical, ECG, echocardiographic and laboratory evaluation 1 week before starting chemotherapy to verify the inclusion/exclusion criteria. cTnI and NT-proBNP samples were collected from enrolled patients before and 24 h after each drug administration. Echocardiographic evaluation was performed at baseline (1 week before starting chemotherapy), every 2 cycles, at the end of chemotherapy, and at 3-, 6- and 12-month follow-up (Figure 1).

The increase in NT-proBNP was defined as *transient* when elevated NT-proBNP levels decreased within the normal range at repeat measurements during NHDC, and *permanent* when elevated NT-proBNP levels were recorded at each measurement.

### End points and statistics

The primary end point for the sample size calculation was the difference between group A (no or transient NT-proBNP elevation) and group B (permanent NT-proBNP elevation) in the percentage change of LVEF from baseline to 1-year follow-up. In accordance with Sandri *et al* (2005), assuming an *α*-error of 0.05 and a power of 90%, the required sample size was 54 patients. Then, the relationship between permanent NT-proBNP elevation throughout NHDC cycles and structural/functional cardiac alterations, both during NHDC and after the end of drug administration, was evaluated. The same relationship was investigated for cTnI.

The percentage Δ (baseline–peak) NT-proBNP was obtained using the following formula: (baseline NT-proBNP−peak NT-proBNP/baseline NT-proBNP) × 100; the highest NT-proBNP measurement was recorded as the peak value reached during NHDC.

Results were expressed as mean±s.d. for normally distributed continuous variables, and as median and interquartile range (IQR; 25th and 75th percentile) for not normally distributed data. Categorical variables were reported as count and percentage. Continuous variables were tested for normal distribution using the Kolmogorov–Smirnov test. Paired and unpaired statistical comparisons were made using the Wilcoxon test and the Student's *t*-test or Mann–Whitney *U*-test, respectively. Categorical variables were compared using *χ*^2^ or Fisher exact test, where appropriate.

The NT-proBNP group, time of assessment, baseline NT-proBNP, peak NT-proBNP, %Δ NT-proBNP (baseline–peak), protocol of therapy, cumulative dose of anthracyclines, age, baseline LVEF, hypertension, diabetes, hypercholesterolemia, smokers, ACE-inhibitors, *β*-blockers, AT_2_-receptor blockers were initially tested by univariate analysis; those with a *P*-value of <0.1 were entered into a mixed linear regression model to assess whether LVEF impairment over time was dependent on the response pattern of NT-proBNP. Results were reported as *β*-coefficient±s.e. and *P*-value.

Receiver operating characteristic (ROC) curve analysis was used to identify any cutoff of %Δ NT-proBNP (baseline–peak) predictive of LV impairment from baseline to 3-, 6- and 12-month follow-up. %Δ (baseline–peak) NT-proBNP was entered into the ROC curve analysis as a continuous predictive variable. LV impairment from baseline to 3-, 6- and 12-month follow-up was used as a classification variable (target event). LV impairment was defined as a decrease in LVEF of ⩾20% and/or an increase in LVESV of ⩾15% from baseline (Mele *et al*, 2006).

Statistical significance was evaluated against 1000 bootstrap samples. A probability value of <0.05 was considered significant. All analyses were performed with the statistical software package SPSS (SPSS Inc., Chicago, IL, USA).

## Results

Demographical, clinical and echocardiographic data and chemotherapy protocol did not differ significantly between groups (Table 1).

Biochemical measurements showed normal NT-proBNP and cTnI levels at baseline in all patients. During NHDC, cTnI was abnormal only occasionally in four patients. Conversely, elevated NT-proBNP levels were detected in many samples, and patients were divided into two groups according to changes in NT-proBNP concentrations: group A (*n*=50) showing normal (*n*=23) or transiently elevated NT-proBNP levels (*n*=27, increase at 24 h with subsequent normalisation) at repeated measurements, and group B (*n*=21) showing persistently elevated NT-proBNP levels at repeated measurements (Table 2). No difference was found between groups in the cumulative dose of anthracyclines.

### Echocardiographic results

During NHDC, only two patients showed a >10% reduction in LVEF, but in both cases LVEF remained >50%. The magnitude of mean LVEF reduction across NHDC was 2% (IQR −0.25% −4%) in group A and 4% (IQR 3% 6%) in group B.

**Figure 2:**
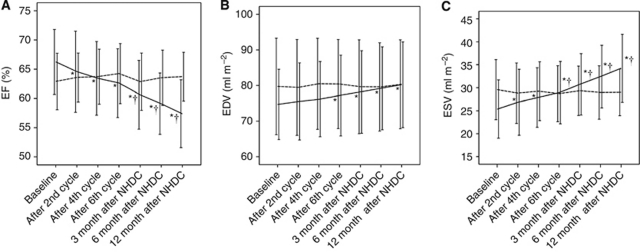
Serial changes in LVEF (**A**), LVEDV (**B**) and LVESV (**C**) in group A (dashed line) and group B (solid line). Standard deviation is also plotted. ^*^P-value (*vs* baseline) <0.05; ^†^*P*-value (A *vs* B) <0.05.

In group A, no significant differences in serial measurements of LVEF were observed, whereas group B showed a significant reduction in LVEF over time (Figure 2A), in particular 3 months after the end of NHDC administration. At mixed linear regression analysis, permanent NT-proBNP elevation during NHDC was correlated with LVEF reduction 12 months after the end of NHDC (*β*=22.8±9.5, *P*=0.013).

Regarding LV volumes, no differences were observed in group A in serial measurements compared with baseline; in group B LVEDV tended to increase significantly after the sixth NHDC cycle, whereas LVESV was found to increase by the second NHDC cycle (Figure 2).

Moreover, LVEF and LVESV were significantly different in group A and group B at 3-month follow-up and at the end of NHDC, respectively (Figure 2).

### ROC curve analysis

**Figure 3:**
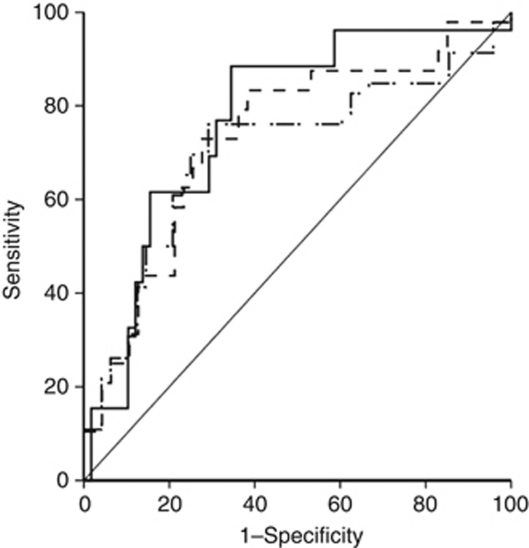
ROC curves: Δ (baseline–peak) NT-proBNP was predictive of LV impairment at 3- (solid line), 6- (dot-dashed line) and 12-month follow-up (dashed line).

Δ (baseline–peak) NT-proBNP was predictive of LV impairment at 3- (AUC=0.81, 95% CI=0.70–0.90), 6- (AUC=0.75, 95% CI=0.62–0.83) and 12-month follow-up (AUC=0.77, 95% CI=0.66–0.86) (Figure 3). A Δ (baseline–peak) NT-proBNP value higher than 36% was predictive of LV impairment at 3- (sensitivity 92.3%, specificity 65.2%), 6- (sensitivity 78.3%, specificity 70.8%) and 12-month follow-up (sensitivity 79.2%, specificity 72.3%) (Figure 3). Patients with persistent NT-proBNP elevation had a Δ (baseline–peak) NT-proBNP value higher than 36% *vs* just 24% in group A (*P*<0.001).

### Clinical events

At 12-month follow-up, two patients experienced dyspnoea (after the end of chemotherapy) with clinical evidence of heart failure, and two additional patients developed supraventricular arrhythmias (during chemotherapy). It is worth noting that these four patients belonged to group B (with persistent NT-proBNP elevation).

## Discussion

There is increasing evidence for the clinical relevance of chemotherapy-induced cardiotoxicity in cancer patients. The large number of cancer survivors increases the likelihood of developing LV dysfunction and overt heart failure. This is especially true in breast cancer patients who need to be treated with anthracyclines, the most cardiotoxic antiblastic drugs (Singal and Iliskovic, 1998; Hequet *et al*, 2004; Yeh and Bickford, 2009). In addition, female gender is a well-established risk factor for cardiotoxicity (Lipshultz *et al*, 1995).

Anthracycline-induced cardiotoxicity has traditionally been categorised into acute, early-onset chronic progressive, and late-onset chronic progressive. It leads predominantly to LV dysfunction and subsequent heart failure, often with dismal prognosis. In the clinical setting, monitoring of anthracycline-induced cardiotoxicity is usually performed by LVEF measurement, as assessed by echocardiography or radionuclide scintigraphy. LVEF depression represents a late marker of cardiotoxicity and allows the identification of patients with irreversible myocardial damage. The need for an early marker of cardiac injury is therefore undeniable, and several studies addressed this topic. In patients with aggressive malignancies treated with HDC, Cardinale *et al* (2000, 2004) demonstrated that cTnI accurately predicts future LVEF depression. This finding was confirmed in subsequent studies that highlighted the value of troponins in this patient subset (Auner *et al*, 2003; Lipshultz *et al*, 2004; Kilickap *et al*, 2005).

In recent years, several studies have suggested the use of BNP as a screening test for asymptomatic LV dysfunction (Latour-Pérez *et al*, 2006; Romano *et al*, 2009), and some authors have reported conflicting results regarding the value of BNP and NT-proBNP in cancer patients undergoing chemotherapy (Bauch *et al*, 1992; Suzuki *et al*, 1998; Meinardi *et al*, 2001, 2002; Sandri *et al*, 2005; Germanakis *et al*, 2006; Kouloubinis *et al*, 2007).

In our study, we evaluated breast cancer patients treated with chemotherapy regimens containing liposomal doxorubicin and epirubicin with a lower degree of cardiotoxic activity than doxorubicin. At present, this treatment is commonly used in breast cancer patients, and our results may have an important clinical impact. To the best of our knowledge, this is the first study evaluating serial determination of cTnI and NT-proBNP in this subset of patients.

Our results show a different response of NT-proBNP in patients undergoing anthracycline-based chemotherapy, with subsequent impairment of LV function only in patients with persistent NT-proBNP elevation. This finding makes the detection of NT-proBNP abnormalities very useful in the early identification of patients at high risk of developing LV dysfunction. As a matter of fact, the persistence of elevated NT-proBNP concentrations may reflect decreased contractile reserve. A recent review has investigated the potential mechanisms underlying anthracycline-induced cardiotoxicity (Sawyer *et al*, 2010). Among the proposed mechanisms, ‘sarcomere disruption’ seems to better support the hypothesis of reduced contractile reserve (increased BNP levels), even in the absence of cell death (normal troponin values) (Mortensen *et al*, 1986; Rowan *et al*, 1988; Mackay *et al*, 1994). Anthracycline exposure induces titin degradation: titin is a giant myofilament protein, which serves as a scaffold for assembly of myofilamentous proteins into sarcomeres. As a consequence, its integrity is essential for the dynamic regulation of contractile function. The loss of intact titin has been demonstrated in failing human hearts, implicating titin disruption in the pathogenesis of progressive ventricular dysfunction (Wang *et al*, 1988; Helmes *et al*, 1996, 2003).

Our results concerning the diagnostic value of cTnI are unexpected, as in the current literature, chemotherapy-induced cTnI elevation is reported in roughly 30% of patients undergoing HDC (Cardinale *et al*, 2000, 2004). Most studies focused on patients undergoing HDC, and only limited information is available about NHDC, leaving the debate open. In 100 consecutive patients receiving low-to-moderate doses of anthracyclines, Dodos *et al* (2008) failed to demonstrate any predictive value of either troponins or BNP. Feola *et al* (2011) did not find any differences in cTnI measured at baseline and 1 month after NHDC between patients developing cardiotoxicity and the remaining population.

Sawaya *et al* (2011) demonstrated that, after 3 months of treatment, elevated cTnI levels were observed in 28% of patients and were predictive of cardiotoxicity. However, they used a high-sensitivity assay that enabled detection of minimal marker elevation.

Our different findings may be due to two factors. On one hand, our study population was treated with lower doses of cytotoxic drugs, causing less myocardial injury. On the other, according to our study protocol, only one blood sample was scheduled after each chemotherapy cycle because all patients were outpatients. Conversely, previous studies on HDC usually enrolled inpatients, in whom serial blood samples were collected, increasing the likelihood of detecting cTnI elevation.

### Comparison with previous studies on natriuretic peptides

Up to now, only few studies with a limited number of patients have evaluated the role of natriuretic peptides after chemotherapy in cancer patients.

Our results are consistent with those reported by Sandri *et al* (2005), who studied 52 patients treated with HDC for aggressive malignancies. They observed three different behaviours of NT-proBNP concentrations: (1) increased NT-proBNP levels up to 72 h after the end of chemotherapy in 17 patients; (2) initial elevated NT-proBNP levels with return to baseline values after 72 h in 19 patients; and (3) no NT-proBNP elevation in 16 patients. The evaluation of LV function showed that only persistently increased plasma NT-proBNP early after HDC was strongly associated with the development of cardiac dysfunction. Our results show a similar pattern of NT-proBNP changes in patients undergoing NHDC, with functional impairment more likely to occur in patients with persistently elevated NT-proBNP levels.

Some studies have evaluated natriuretic peptides in NHDC patients, showing elevated BNP or NT-proBNP levels in most subjects (Meinardi *et al*, 2001; Pichon *et al*, 2005; Kouloubinis *et al*, 2007; Feola *et al*, 2011). However, no assessment of their predictive value in identifying patients at high risk of developing LV dysfunction and/or heart failure was made at subsequent follow-up. In the study by Feola *et al* (2011), the patients who experienced cardiotoxicity showed elevated BNP levels 1 and 2 years after NHDC. However, no significant BNP changes were observed over time, which is more clinically relevant for the early identification of patients at high risk of developing LV dysfunction. Conversely, the results of our study demonstrate that NT-proBNP may serve as a useful marker for the early detection of such patients.

### Study limitations

This study has some limitations that need to be considered. First, it is an observational study, not a randomised, controlled trial. Second, the collection of only one blood sample 24 h after each NHDC cycle may have led to a low number of ‘positive’ patients, especially cTnI-positive patients. Data from previous studies on HDC show that ∼20% of patients have abnormal values 36 or 72 h after treatment. Notwithstanding this, the results obtained with NT-proBNP are not affected by this limitation, because the most important finding is the return to baseline values in the sample collected before the subsequent cycle of chemotherapy (14 or 21 days later). In addition, we also enrolled patients with arterial hypertension, although this condition is known to cause NT-proBNP elevation. This may mask or reduce the negative effects of chemotherapy on LV volumes and function.

However, our inclusion/exclusion criteria allowed us to enrol a study population that was more representative of the ‘real world’. Our data showed normal NT-proBNP levels at baseline also in hypertensive patients, and no difference was present in the frequency of hypertension or antihypertensive drugs between the two groups of patients divided according to NT-proBNP behaviour.

Finally, no assessment of diastolic function was made, thus limiting the possibility of investigating whether cardiac biomarkers can predict diastolic impairment of LV function.

### Clinical implications

Antineoplastic therapy is frequently complicated by the development of cardiotoxicity, which leads to cardiomyopathy evolving towards heart failure. In some instances, LV impairment is detected too late at echocardiography, when significant myocardial damage has already occurred. The availability of a biomarker for the identification of patients at high risk of developing cardiotoxicity would be of particular benefit, prompting oncologists and cardiologists to ensure close monitoring of these patients and to start adequate preventive or therapeutic management of LV dysfunction.

Serial measurements of NT-proBNP levels in NHDC patients may be even more useful than cTnI in the early identification of patients at high risk of developing anthracycline-induced cardiotoxicity. As recently demonstrated by Cardinale *et al* (2010), LVEF recovery in cancer patients undergoing anthracycline administration may be achieved if LV dysfunction is detected early and appropriate heart failure therapy is promptly initiated.

Further studies are warranted to test the possibility of reducing anthracycline-induced cardiotoxicity by adopting an NT-proBNP-guided preventive strategy.

## Figures and Tables

**Table 1:** Characteristics of the study population

	**Overall (*n*=71)**	**Group A (*n*=50)**	**Group B (*n*=21)**	***P*-value**
Age (years)	54.0±11.7	52.6±12.1	57.3±10.2	0.124
BMI (kg m^–2^)	27.5±5.5	28.0±5.9	26.2±3.9	0.209
Hypertension	25 (35.2%)	18 (36.0%)	7 (33.3%)	0.830
Diabetes mellitus	6 (8.5%)	6 (12.0%)	0	0.170
Hypercholesterolemia	62 (87.3%)	44 (88.0%)	3 (61.9%)	1.000
Smokers	25 (35.2%)	15 (30.0%)	10 (47.6%)	0.156
Baseline SBP (mm Hg)	130±14	132±16	126±9	0.145
Baseline DBP (mm Hg)	82±7	83±7	81±5	0.292
Baseline HR (b.p.m.)	74±10	75±11	72±8	0.161
Baseline LVEF (%)	64±11	64±5	66±6	0.147
Baseline LVEDV (ml m^–2^)	77±12	77±12	75±10	0.386
Baseline LVESV (ml m^–2^)	27±7	28±6	25±6	0.155
				
*Therapy*				
FEC protocol	37 (52.1%)	26 (52.0%)	11 (52.4%)	0.977
Liposomal doxorubicin	34 (47.9%)	24 (48.0%)	10 (47.6%)	0.977
ACE-inhibitors	18 (25.4%)	14 (28.0%)	4 (19.0%)	0.429
Aspirin	37 (52.1%)	26 (52.0%)	11 (52.4%)	0.977
AT_2_-receptor blockers	12 (16.9%)	8 (16.0%)	4 (19%)	0.740
*β*-Blockers	8 (11.3%)	7 (14.0%)	1 (4.8%)	0.221

Abbreviations: BMI=body mass index; SBP=systolic blood pressure; DBP=diastolic blood pressure; HR=heart rate; LVEDV=left ventricular end-diastolic volume; LVEF=left ventricular ejection fraction; LVESV=left ventricular end-systolic volume; FEC=fluorouracil, epirubicin, cyclophosphamide; ACE=angiotensin-converting enzyme; AT=angiotensin.

**Table 2:** NT-proBNP (ng l^–1^) levels before and 24 h after NHDC (values are expressed as median value (25th–75th percentile)

	**Pre-NHDC**	**24 h after NHDC**	***P*-value**
*Group A (*n=*50)*			
1st NHDC cycle	44 (25–85)	127 (93–205)	<0.001
2nd NHDC cycle	58 (36–98)	145 (114–216)	<0.001
3rd NHDC cycle	64 (34–118)	138 (101–216)	<0.001
4th NHDC cycle	76 (34–116)	152 (108–200)	<0.001
5th NHDC cycle	78 (45–102)	153 (108–218)	<0.001
6th NHDC cycle	82 (52–116)	138 (110–201)	<0.001
			
*Group B (*n=*21)*			
1st NHDC cycle	87 (68–150)	300 (227–356)	<0.001
2nd NHDC cycle	252 (221–282)	344 (280–406)	<0.001
3rd NHDC cycle	244 (222–292)	356 (312–447)	<0.001
4th NHDC cycle	250 (232–323)	341 (315–408)	0.004
5th NHDC cycle	244 (218–280)	371 (322–394)	<0.001
6th NHDC cycle	241 (229–293)	370 (278–417)	<0.001

Abbreviations: NHDC=not-high-dose chemotherapy; NT-proBNP=N-terminal pro-brain natriuretic peptide.
